# Topical Application of Chinese Formula Yeliangen Promotes Wound Healing in Streptozotocin-Induced Diabetic Rats

**DOI:** 10.1155/2022/1193392

**Published:** 2022-11-29

**Authors:** Abdulbaset Al-Romaima, Xiong Guan, Xihui Qin, Yinan Liao, Guiming Qin, Shixiong Tang, Jie Feng

**Affiliations:** ^1^School of Pharmaceutical Science, Guangxi Medical University, Nanning, 530021 Guangxi, China; ^2^The Eighth People's Hospital of Nanning, Nanning 530007, China; ^3^The Second Affiliated Hospital of Guangxi Medical University, Nanning, 530007 Guangxi, China

## Abstract

Diabetic wound is one of the most severe complications of diabetes mellitus (DM). Despite the associated risks of wound healing impairment in diabetes, treatment strategies remain limited. Yeliangen (YLG) is a Chinese formulation mainly composed of the rhizome of *Coptis chinensis*, the root of *Isatis tinctoria*, and the leaf of *Isatis indigotica*. We investigated the wound healing effects of YLG in type 2 diabetic (T2DM) rats, which were induced by intraperitoneal administration of streptozotocin after a high-fat diet for four weeks. 3 × 3 cm^2^ full-thickness excisional wounds were created on the dorsal surface of rats and then divided to control (DC), negative (DPJ), positive (DPC), and YLG-treated (DYLG) groups. Rat's wounds were treated twice daily for 21 days. Wound area and wound contraction were detected on days 0, 3, 7, 14, and 21. Histopathological examinations were performed by H&E staining and immunohistochemistry (IHC). The biochemical parameters, mRNAs, and protein expressions were analyzed through enzyme-linked immunoassays (ELISA), qPCR, and western blot, respectively. Compared with other groups, the histological changes of wound tissue in the DYLG group were improved, and the expressions of CD31, eNOS, and PCNA were significantly upregulated. Besides, YLG significantly reduced the inflammatory factors' expressions of TNF-*α*, NF-*κ*B, MMP-9, and IL-1B on days 7, 14, and 21 postwounding. Moreover, YLG induced angiogenesis and neovascularization by significantly increasing the levels of VEGF, TGF-*β*1, EGF, PDGF, and SDF-1*α* on days 3, 7, and 14. In conclusion, YLG improved wound healing by reducing inflammation and increasing angiogenesis which may provide an alternative and effective approach for diabetic wound therapy.

## 1. Introduction

Diabetes mellitus (DM) is a metabolic disorder characterized chiefly by chronic hyperglycemia as a result of insulin secretion deficiency or insulin resistance [1]. Diabetes prevalence is estimated to reach 9.3% (463 million people) worldwide in 2019, maybe increasing to 10.2% (578 million) by 2030 and 10.9% (700 million) by 2045, and more than 90% are type 2 diabetic (T2DM) [2]. Diabetes is generally considered as one of the main factors affecting the wound healing process [3]. Globally, the development of diabetes has been accompanied by an increase in the incidence of diabetic ulcers, which are susceptible to infection, leading to increased morbidity and mortality, a considerable financial burden on the patient, and eventually amputation [4–6]. In several complications of diabetes, inflammation, neuropathy, and vascular disease are the main factors of delayed wound healing. Moreover, most diabetic patients develop diabetic neuropathy, loss of lower limb pain, vascular ischemia, and various metabolic abnormalities, leading to unknown wounds until serious infection [7, 8].

The biological process of wound healing goes through different stages, determined by their roles, for example, hemostasis, inflammation, proliferation (angiogenesis), remodelling, and epithelialization [9–11]. The first stage of the cellular repair process involves activating platelets, aggregating, and adhering to harmless endothelial cells, known as clotting, to maintain hemostasis. Compared with normal subjects, some changes can be observed during the hemostasis phase in diabetic wounds, with decreased hypercoagulability and fibrinolysis [9]. The inflammatory phase occurs when neutrophils, macrophages, and mast cells produce inflammatory cytokines, such as interleukin 1B (IL-1B), interleukin 6 (IL-6), and tumour necrosis factor *α* (TNF-*α*) that cause tissue damage. These cytokines are out of balance in diabetic patients, leading to changes in wound repair [10]. When the inflammation subsides, the proliferation phase takes place, in which angiogenesis restores oxygen supply and production of extracellular matrix (ECM) proteins, including collagen, fibronectin, connectin, and keratinocyte migration. All of these are necessary to ensure the integrity and functionality of the organization [11]. During the remodelling phase, the collagen synthesized is larger than the degraded, replacing the intermittent outer matrix which is initially produced by fibrin and fibronectin.

Angiogenesis is an essential biochemical mechanism for wound healing and plays a crucial role in wound repair [12]. It is a complex, multistep process in which endothelial cells develop from parent blood vessels and then migrate, proliferate, and anastomize with other blood vessels, leading to regeneration, development, and homeostasis. DM has been studied to inhibit angiogenesis [13]. Besides, systemic microangiopathy can reduce the nutritional supply of ulcer tissue, leading to impaired healing of diabetic foot ulcers [14]. So angiogenesis is very important in diabetic patients with ulcers. A previous study reported that DM was a main cause of the impairment in wound healing due to prolonged inflammatory periods, defective angiogenesis, and reduced fibroblast proliferation [15]. Patients with diabetes induce wound closure defects by altering fibroblasts. Contraction with the initial stage of wound healing is necessary for the formation and reepithelialization of granulation tissue. Fibroblasts play an essential role in wound contraction by activating collagen production, which means the occurrence of fibroblasts at the wound site is a feature of tissue contraction [16].

Growth factors are involved in the normal healing process and play a vital role in wound healing. Growth factors, including vascular endothelial growth factor (VEGF), transforming growth factor (TGF), platelet-derived growth factor (PDGF), and epidermal growth factor (EGF), promote wound healing through their physiological effects. Growth factors affect the proliferation and migration of a variety of cells, stimulation of endothelial cells, angiogenesis, and chemotaxis of fibroblasts and inflammatory cells, ranging from chronic, nonhealing wounds to hypertrophic scar or keloid formation [17–19].

Moreover, inflammation plays a vital role in delayed wound healing. The cellular signalling protein TNF-*α* is involved in the systemic inflammatory response associated with the destruction of connective tissue. Inhibition of TNF-*α* plays a crucial role in tissue repair. Therefore, controlling TNF-*α* expression is significantly helpful for diabetic wound healing. Interleukin (IL) is an essential inflammatory cytokine produced by blood monocytes and tissue macrophages [20]. Elevated IL levels are associated with the development of insulin resistance and abnormal healing in diabetes. IL levels increase in patients with diabetic foot ulcers and decrease with ulcer healing [19].

Various therapeutic modalities have been reported, and there were controversial results in wound healing in DM patients [21]. So far, many preclinical animal studies focus on a single therapy, including the growth factor (VEGF), antibiotic therapy, the HMG-CoA reductase inhibitors (such as atorvastatin), debridement, laser treatment, and herbal medicine. There is no specific effective medicine for the treatment of diabetic wounds because of the multifactorial problem of chronic wounds in diabetes. In addition, until recently, research on diabetic wound healing still focused on the initial stages rather than preventing diabetic wound healing.

At present, plant medicine has been widely used in standard wound care practice and rapid evidence-based clinical studies, and it is an essential resource for developing new medicines to treat diabetes-related diseases and other diseases [22]. Traditional Chinese medicine (TCM) is a significant pharmaceutical resource with a long history and has great potential in the development of pharmacology. In recent years, TCM has been widely utilized as an alternative to mainstream treatments for different diseases. *Astragalus membranaceus* and *Rehmannia glutinosa*, the two main components of TCM, can promote the proliferation of fibroblasts, which is the main step of wound healing [23, 24]. The rhizome of *Coptis chinensis* Franch. (Ranunculaceae family) is a plant used in TCM which contains alkaloids as the main compounds, including berberine, an alkaloid that is known to have prominent antimicrobial and anti-inflammatory properties, coptisine, epiberberine, jateorhizine, and palmatine [25]. *C. chinensis* has antioxidant effects, including scavenging oxygen-free radicals, reducing lipid peroxidation, and enhancing antioxidant enzyme activity. It is a potential drug for the prevention and treatment of diabetes and its complications [25]. The root of *Isatis tinctoria* L. (Cruciferae family) has been used medically for thousands of years, containing abundant indole alkaloids such as indigotin, isatin, inderubin, isaindigodione, hydroxyindirubin, indicant, gingdainone, and tryptanthrin, and shows good antibacterial activity [26, 27]. The leaf of *I. indigotica* Fort. (Cruciferae family) is an official Chinese medicine for the treatment of infection and inflammation. It is traditionally used as an antiviral, antibacterial, anti-endotoxin, and immunomodulatory agent [28], and indigotin, isatin, and indole-3-formaldehyde are the main chemical compositions [29]. Currently, it has been confirmed that an ointment prepared from the extract of *Quercus infectoria* gall accelerated wound healing in a diabetic mice model by shortening the inflammatory phase, inducing apoptosis, increasing the expression of Bcl-2 and p53 mRNA, possessing antioxidant properties, and promoting cellular proliferation [30]. Rutin, an antioxidant flavonol, can induce apoptosis in rats with endometriosis by modulating Bcl-2, Bax, and caspase levels and boosting antioxidant expressions [31]. In a rat model, it has been established that the topical utilization of *Trifolium repens* ointment promoted wound healing by increasing angiogenesis, fibroblast, fibrocyte, mast cell distribution, and apoptosis-related gene regulation [32].

Impairment of wound healing is one of the main complications of diabetes, and despite the associated risks, treatment strategies for diabetic wounds stay limited, and there is no specific effective drug till now. This may be due to an incomplete understanding of the underlying pathological mechanisms [33]. More studies, such as treatments and interventions, are required to understand the different stages of wound healing in diabetes, to help unhealed wounds reach a state of health and integrity. In this study, we prepared a Chinese herbal formulation (YLG) composed mainly of *C. chinensis*, *I. tinctoria*, and *I. indigotica* as the main formula composition, and its aqueous extract effects on wound healing in the T2DM rat model have been investigated.

## 2. Materials and Methods

### 2.1. Preparation of the YLG Formulation

The Chinese formulation is mainly composed of the rhizome of *C. chinensis*, root of *I. tinctoria*, and the leaves of *I. tinctoria*. The dry material was pulverized. The extraction process was as follows: 1000 g of crude dried powder was extracted with 4 l of distilled water under reflux three times (20 minutes each time) to obtain the filtrate. The filtrate was then combined and vacuum recovered under reduced pressure to obtain the crude extract. After that, the crude extract was dried using a freezing dryer to get 175 g. Using petroleum jelly (PJ) as the base, the dried extract was mixed with PJ (extract: PJ = 1 : 10) and heated and stirred evenly to make the ointment, named YLG. Then, it was sealed and kept in the refrigerator at 4°C and applied later on the rats' wounds twice a day.

### 2.2. Animals

Healthy specific pathogen-free (SPF) male Sprague-Dawley (SD) rats, weighing 200–250 g, were purchased from the Animal Center of Guangxi Medical University (SYXK Gui 2014-0003, Guangxi, China). All animals were treated according to the animal ethics requirements of Guangxi Medical University. They were housed under standard conditions, fed on a standard diet and water ad libitum, and maintained under a 12 h/12 h light-dark cycle and 25 ± 2°C temperature.

### 2.3. T2DM Model Induction

Ninety-six SD rats were fed a high-fat diet (HFD, 60% fat calories, and 70% animal fat) until the rat weight reached 330 ± 20 g, and then, low dose (30 mg/kg) of streptozotocin (STZ, Beijing Solarbio Science & Technology Co., China; dissolved in citrate buffer of pH 4.5) was intraperitoneally injected (i.p.) into the abdominal cavity. On the seventh day morning, blood was collected from the tails of rats to determine fasting blood glucose levels. Rats with fasting (12 h) blood glucose value ≥ 13.6 mmol/l were considered T2DM model animals with successful modelling, which were included in the experimental range [28].

### 2.4. Diabetic Wound Model

The T2DM rats were anaesthetized by i.p. administration of pentobarbital (40 mg/kg). The dorsal hair of rats was shaved, and the skin was cleaned with 70% ethanol. A 3 × 3 cm^2^ full-thickness open excision wound was made on the loose subcutaneous tissue with a sterilized surgical scalpel blade. The incision was carried out under aseptic conditions. When recovering from anaesthesia, the rats were placed in sterile cages, and YLG treatment was started the next day. To prevent rats from harming their own wounds, skin wounds were formed in the back region, and each rat was kept in a separate sterile cage.

### 2.5. Experimental Design

After excision skin wound was created on the back of T2DM rats, they were equally divided into four groups (*n* = 24 per group), i.e., (i) diabetic control group (DC): the rats did not receive any medicine; (ii) diabetic negative control (DPJ): the rats received PJ topically; (iii) diabetic positive control (DPC): the rats received iodine topically; and (iv) diabetic-treated group (DYLG): the rats received YLG topically. The rats were treated twice daily. For each experimental time point (3^rd^, 7^th^, 14^th^, and 21^st^), six rats were taken from each group every time point to carry out the experiment.

### 2.6. General and Serum Biochemical Indexes of T2DM Rats Were Observed or Detected

- Mental activity, hair, food and water intake, urine and faeces, death, and wound infection of T2DM rats were observed during the experiment
- On days 0, 3, 7, 14, and 21, the rat's body weights were detected, and the levels of fasting blood glucose (FBG) in rats were measured using a glucose meter (ACCU-CHEK, Roche Diagnostics GmbH, Mannheim, Germany)
- The rat's blood was collected on days 0, 3, 7, 14, and 21 and then centrifuged at 3000 g for 15 minutes to get the serum, respectively. The levels of insulin, C-peptide, NF-*κ*B, and IL-6 in the serum rats were determined by ELISA kit and measured according to the manufacturer's instructions (Elabscience Biotechnology Co., China)

### 2.7. Measurement of the Wound Area and the Wound Contraction of the Rats

On days 0, 3, 7, 14, and 21, postwounding, the progressive change in wound area was determined using a digital camera (Nikon, Japan). The wound area was calculated planimetrically from photographic pictures using ImageJ analysis software (ImageJ, NIH, USA). The following formula was used to detect the wound closure ratio:

(1) $$\begin{array}{c} \text{Wound closure}\%=\frac{0\text{ day wound area}-\text{particular day wound area}}{0\text{ day wound area}}\times 100\%. \end{array}$$

### 2.8. Tissue Harvesting

On days 3, 7, 14, and 21, six rats were anaesthetized by i.p. injection with pentobarbital (40 mg/kg) for each group. The blood was collected from the dorsal aortic vein and put in a 5 ml centrifuge tube and then centrifuged to get supernatant which was kept at –20°C and used later for ELISA biochemical assay. Six rats of each group were taken at every experimental time point, and the entire wounded skin with a margin of approximately 5 mm^2^ of surrounding unwounded skin was excised. These tissues were divided into three portions. One portion was fixed in 10% buffer formalin for 48 h and embedded in paraffin for histopathological and IHC analysis. The second portion was put in EP tube, stored at –80°C until RNA extraction, and used for RT-qPCR analysis. The third portion was also put in an EP tube, stored at –20°C, and used for western blotting analysis.

### 2.9. Haematoxylin and Eosin (H&E) Staining

5 *μ*m thick slices of paraffin-embedded skin tissues were cut, deparaffinized in xylene, and then rehydrated in graded concentrations of ethanol. Sections were mounted on glass slides and stained with H&E according to the standard procedure. The sections were inspected, and images were captured using a laser scanning confocal microscope (Olympus, Tokyo, Japan).

### 2.10. Immunohistochemical Examination

Skin tissues were cut and disposed of as step 2.9. The endogenous peroxidase activity in the skin sections was inhibited by hydrogen peroxide (0.1%). The sections were blocked with bovine serum for 30 min, then incubated with anti-EGF polyclonal antibody CD31 (1 : 2000), endothelial nitric oxide synthase (eNOS) (1 : 100), and proliferating nuclear antigen (PCNA) (1 : 200), for 12 hr, and after that incubated with a secondary antibody for one hour. After that, we counterstained the sections with haematoxylin. The microscopic examination images were recorded using a laser scan confocal microscope (Olympus BX53 Light/Fluorescence Microscope). The expression of CD3, PCNA, and eNOS was quantified and measured by using ImageJ Fiji from https://imageJ.nih.gov/ij/ (NIH, USA).

### 2.11. Quantitative Polymerase Chain Reaction (qPCR)

The mRNA expressions of VEGF, EGF, TGF-B1, SDF-1, IL-10, IL-1B, TNF-*α*, NF-*κ*B, and MMP-9 were detected using qPCR. RNA was extracted from wound tissues using AxyPrep Multisource Total RNA Miniprep Kit (Axygen Scientific, Union City, CA, USA). Then, cDNA was synthesized using 5× Primescript cDNA synthesis kit (Takara, Japan). After that, cDNA was used as a template for the subsequent RT-PCR. PowerUp SYBR Green Master Mix (Thermo Fisher Scientific, USA) was used to carry out the RT-PCR assay in 7300 Real-Time PCR System (Thermo Fisher Scientific, USA). The forward (F) and reverse (R) primers and probes were as follows: *β*-actin (F: 5′-CTGAGAGGGAAATCGTGCGTGAC-3′; R: 5′-AGGAAGAGGATGCGGCAGTGG-3′); TNF-*α* (F: 5′-AAAGGACACCATGAGCACGGAAAG-3′; R: 5′-CGCCACGAGCAGGAATGAGAAG-3′); NF-*κ*B (F: 5′-TGTGGTGGAGGACTTGCTGAGG-3′; R: 5′-AGTGCTGCCTTGCTGTTCTTGAG-3′); MMP-9 (F: 5′-CTCCTGGTGCTCCTGGCTCTAG-3′; R: 5′-GCTGTGTGTCCGTGAGGTTGG-3′); IL-1B (F: 5′-TGTTTCCCTCCCTGCCTCTGAC-3′; R: 5′-CGACAATGCTGCCTCGTGACC-3′); VEGF (F: 5′-GTGACAAGCCAAGGCGGTGAG-3′; R: 5′-GATGGTGGTGTGGTGGTGACATG-3′); TGF-*β*1 (F: 5′-GACCGCAACAACGCAATCTATGAC-3′; R: 5′-CTGGCACTGCTTCCCGAATGTC-3′); EGF: F: 5′-CCATGCTGTTCTCGCTCACCTTC-3′; R: 5′-GTTCCTGGTCTGCTGTGCTGTG-3′); IL-10 (F: 5′-CTGCTCTTACTGGCTGGAGTGAAG-3′; R: 5′-TGGGTCTGGCTGACTGGGAAG-3′); and SDF-1 (F: 5′-TATCTCGGCGGCGTCACCAG-3′; R: 5′-AAACCATCGCTGCGTAGACACTG-3′). The qPCR experiment was performed according to the manufacturer's instruction, and the following thermal cycling profile was used (40 cycles): 50°C for 2 min, 95°C for 10 min, 95°C for 15 s, and 60°C for 1 min. The *^ΔΔ^*Ct method of relative quantification was used to determine fold change in expression and was obtained as 2^-∆∆Ct^.

### 2.12. Western Blotting

The protein expression of VEGF, TGF-*β*1, PDGF, MMP-9, Collagen IV, EGF, and *β*-actin was measured by western blotting. Proteins were extracted by grinding the tissues using a mortar and pestle in liquid nitrogen. Proteins were separated by electrophoresis on 10% sodium dodecyl sulfate-polyacrylamide gels and then transferred to polyvinylidene difluoride (PVDF) membranes (Thermo Fisher Scientific, Millipore, MA, USA). The membranes were then incubated with rabbit polyclonal antibodies against VEGF (1 : 1000), TGF-*β*1 (1 : 1000), PDGF, Collagen IV (1 : 4000), EGF (1 : 1000), and MMP-9 (1 : 1000) for 12 h at 4°C on the shaker. After washing, membranes were incubated with fluorescence-labelled anti-rabbit immunoglobulin G (1 : 10000) for 1.5 h on the shaker at room temperature. The blots were subsequently scanned using an Odyssey Infrared Imaging System v3.0.16 (LI-COR Biosciences, NE), and the band intensity was quantified by ImageJ software (NIH, MD, USA). The reagents and kit numbers are provided as a supplementary table (Table S1).

### 2.13. Statistical Analysis

Experimental results are expressed as mean ± standard error (SEM). Tukey's test of one-way analysis of variance (ANOVA) was used to analyze the significant differences between the groups, and *p* < 0.05 indicated significant differences. All statistical analyses were performed using SPSS 25 software.

## 3. Results

### 3.1. Effects of YLG on General Condition, Body Weight, Fasting Blood Glucose, and Biochemical Indexes of T2DM Rats

The experimental observation revealed that after the success of T2DM modelling, the rats gradually showed different degrees of retarded reaction, lethargy, reduced activity, and yellowing and darkening of fur. Diabetes symptoms such as polydipsia, polyphagia, and polyuria were observed. With the progress of intervention treatment, the administration group had more activities and a good mental state. On days 3, 7, and 14, all rats' weight continued to increase, but on day 21, the weight of rats in the DYLG group was significantly reduced compared to DC, DPJ, and DPC groups (*p* < 0.05) (Table 1).

As shown in Table 2, although, on days 0, 3, 7, and 14, there were no significant differences in blood glucose levels between the diabetic groups, the blood glucose level of rats in the DYLG group was significantly decreased on day 21 (*p* < 0.05), compared with the rats in the DC, DPJ, and DPC groups.

Compared with DC, DPJ, and DPC groups, the insulin level in the DYLG group significantly increased on days 14 and 21 (*p* < 0.05); conversely, there was no statistically significant difference between the groups on days 3 and 7 (*p* > 0.05), as shown in Table 3. There was no statistically significant difference in C-peptide level in the diabetic group on days 0, 3, 7, and 14 (*p* > 0.05), but the C-peptide level in the DYLG group was significantly lower than that in the DC, DPJ, and DPC groups on day 21 (*p* < 0.05) (Table 4).

Blood NF-*κ*B level in the DYLG group was significantly lower than those in the DC group on day 3 (*p* < 0.05) and significantly lower than those in the DC, DPC, and DPJ groups on day 7, 14, and 21 (*p* < 0.05) (Table 5).

On day 0, although there was no significant difference in IL-6 levels between the treated and untreated groups, blood IL-6 levels in all diabetic groups increased. Compared with DC, DPC, and DPJ groups, the level of serum IL-6 in the DYLG group significantly decreased on days 7, 14, and 21 (*p* < 0.05) (Table 6).

### 3.2. Effects of YLG on the Wound Area and Wound Contraction Rate

**Figure 1:**
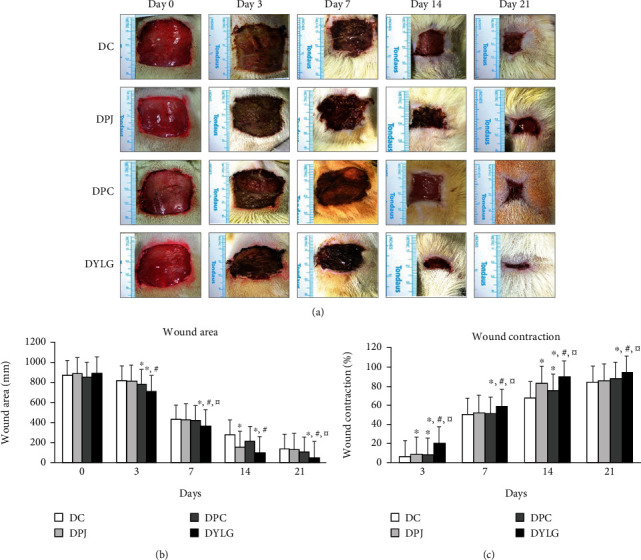
Effect of YLG on the wound area and rate of wound contraction in T2DM rats. (a) Representative wound photographs of groups of DC, DPJ, DPC, and DYLG on days 0, 3, 7, 14, and 21. (b) Changes in wound area of T2DM rats. (c) Change in the wound contraction rate of the rats. DC: diabetic control group; DPJ: diabetic negative control group; DPC: diabetic positive control group; DYLG: diabetic Yeliangen-treated group. ^∗^*p* < 0.05 as compared to DC group, #*p* < 0.05 as compared to DPC group, and *^¤^p* < 0.05 as compared to DPJ group. The results were represented as mean ± SEM. ^∗^The meaning of the symbols in the following figures is the same in this study, so they will not be repeated.

The influence of each treatment group on each stage of wound healing is shown in Figure 1. Compared with the rats in DC, DPJ, and DPC groups, the wound area of rats in DYLG group was significantly reduced on days 3, 7, 14, and 21 (*p* < 0.05), as shown in Figures 1(a) and 1(b). Compared with DC, DPJ, and DPC groups, the wound contraction rate of rats in DYLG group was significantly increased (*p* < 0.05), as shown in Figure 1(c).

### 3.3. H&E Staining Effect of YLG on the Wound of Rats

**Figure 2:**
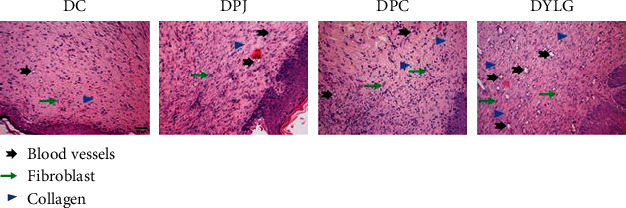
Representative images of haematoxylin and eosin (H&E) staining on the day 21 postwounding in skin wound section of T2DM rats DC, DPJ, DPC, and DYLG groups (20x magnification and scale bar 50 *μ*m).

Figure 2 shows H&E staining images of rat tissue wounds. Compared with the other three groups, DYLG consisted of more fibroblasts and fewer inflammatory cells. DYLG demonstrates well-formed granulation tissue with collagen deposition, which is surrounded by newly formed epithelium. The number of blood vessels increased significantly in the DYLG treatment group. On the other hand, there were still significant inflammatory cells in the DC, DPJ, and DPC groups.

### 3.4. IHC Staining for Expressions of CD31, eNOS, and PCNA

**Figure 3:**
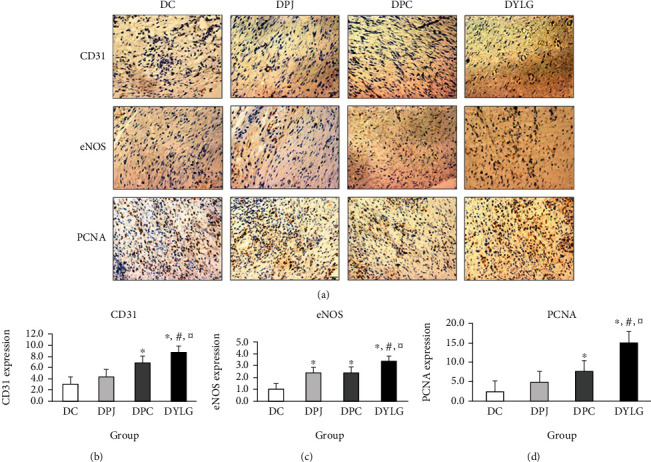
(a) Representative immunohistochemical CD31-, eNOS-, and PCNA-stained wound sections in T2DM groups (DC, DPJ, DPC, and DYLG) (40x magnification and scale bar 50 *μ*m). (b–d) Representative of the semiquantitative expression of CD31, eNOS, and PCNA, respectively, using ImageJ Fiji.

On day 21 postwounding, the formation of new blood vessels at the wound site was detected by CD31 immunostaining (Figure 3(a)). In the DYLG group, the neovascularization was evenly distributed in the granulation tissue, with an obviously vascular lumen and larger circumference. Semiquantitative analysis showed that the expression level of CD31 in DYLG treatment rats was significantly increased compared with DC, DPJ, and DPC rats (*p* < 0.05) (see Figure 3(b)).

The expression of eNOS was detected by IHC staining (Figure 3(a)). Compared to DC, DPJ, and DPC groups, the level of eNOS was significantly increased in the DYLG group (Figure 3(c)). As the fibroblasts and endothelial cells, there was a significant increase in DYLG treatment group compared with the DC, DPJ, and DPC groups. This result suggested that YLG can significantly upregulate the expression of eNOS.

IHC staining was used to verify the expression level of PCNA at the wound edge (Figure 3(a)). The staining results showed that the level of PCNA was significantly increased in DYLG compared with DC, DPJ, and DPC groups (*p* < 0.05) on day 21 postwounding, especially fibroblasts in basal epidermis and subcutaneous layer (Figure 3(d)). These results suggested that YLG increased cell proliferation and promoted wound healing.

### 3.5. YLG Can Reduce the Expression of Inflammatory Cytokines mRNA

**Figure 4:**
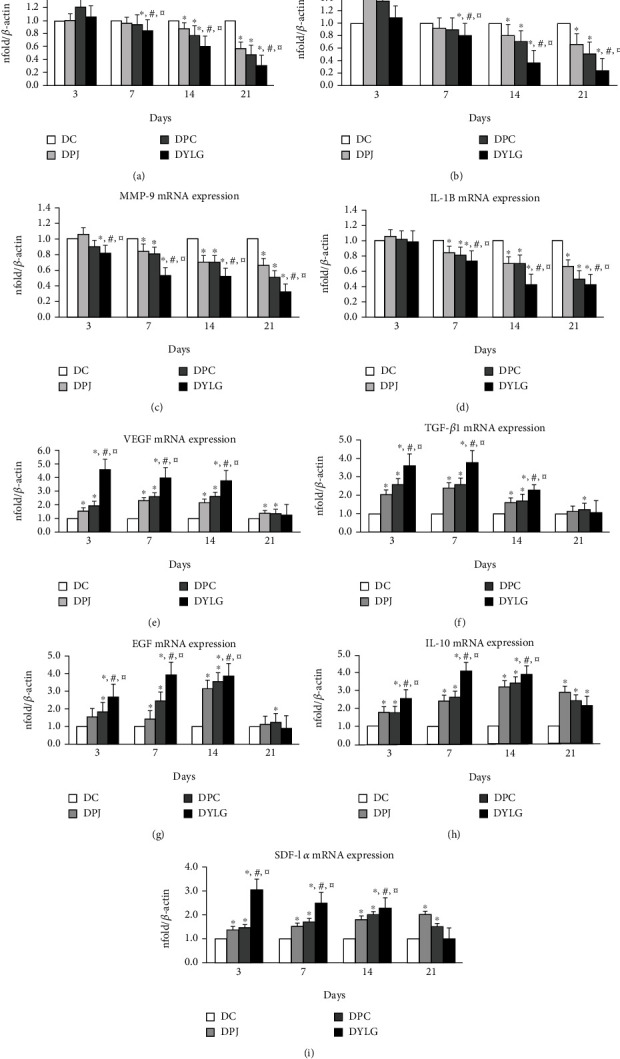
(a–i) Relative mRNA expressions of TNF-*α*, NF-*κ*B, MMP-9, IL-1B, VEGF, TGF-*β*1, EGF, IL-10, and SDF-1*α*, respectively, in diabetic rats on days 3, 7, 14, and 21 postwounding. Data were normalized by *β*-actin at each time point.

Inflammatory factors were increased in all groups in the first three days postwounding. As shown in Figures 4(a)–4(d)), the mRNA expression levels of TNF-*α*, NF-*κ*B, MMP-9, and IL-1B in all groups were increased on day 3 postwounding. However, the mRNA expression levels of TNF-*α*, MMP-9, and IL-1B in the DYLG group were significantly lower than those in DC, DPJ, and DPC groups (*p* < 0.05), and the mRNA expression of NF-*κ*B was slightly decreased, but there was no statistical difference (*p* > 0.05). Compared with DC, DPJ, and DPC, the mRNA expressions of TNF-*α*, NF-*κ*B, MMP-9, and IL-1B in the DYLG treatment group were significantly decreased on days 7, 14, and 21 (*p* < 0.05).

### 3.6. YLG Can Increase the mRNA Expression of Angiogenic Factors VEGF, TGF-*β*1, EGF, IL-10, and SDF-1*α*

We used RT-qPCR to test whether YLG induced neovascularization and angiogenesis by increasing the expression of angiogenic factors. As shown in Figures 4(e)–4(i), the mRNA levels of VEGF, TGF-*β*1, EGF, IL-10, and SDF-1*α* in the DYLG group were significantly increased compared with those in DC, DPJ, and DPC groups on days 3, 7, and 14 (*p* < 0.05). Meanwhile, DYLG group mRNA expression of angiogenic factors decreased on day 21.

### 3.7. YLG Increased the Protein Expression of VEGF, TGF-*β*1, PDGF, EGF, and Collagen IV and Decreased the Protein Level of MPP-9

**Figure 5:**
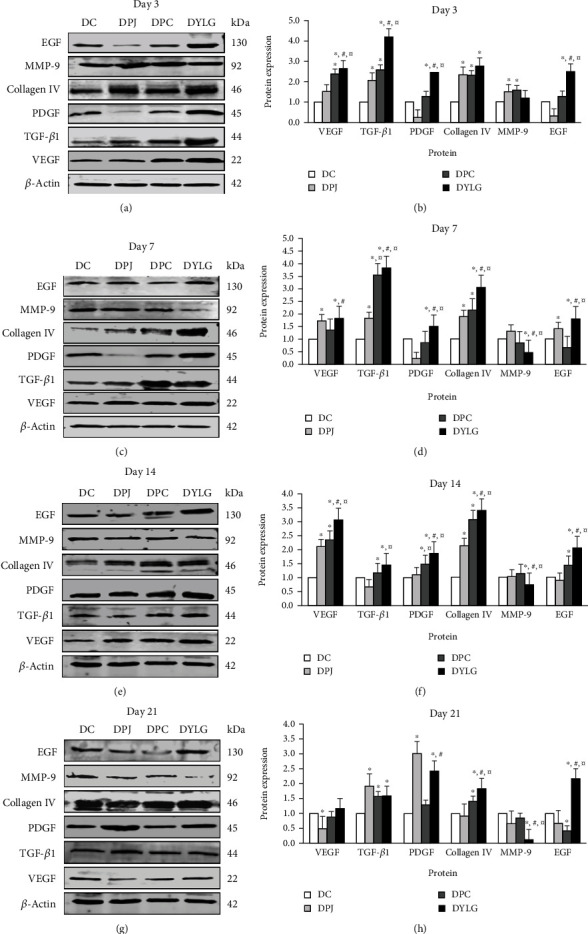
Effect of YLG on protein expressions of VEGF, TGF-*β*1, PDGF, Collagen IV, MMP-9, and EGF in T2DM rats on days 3, 7, 14, and 21 postwounding.

As shown in Figures 5(a) and 5(b), on day 3, the protein expressions of VEGF, TGF-*β*1, PDGF, EGF, and Collagen IV in the DYLG group were significantly higher than those in DC, DPJ, and DPC groups (*p* < 0.05). At the same time, there was no significant difference in the protein expression of MMP-9 among the four groups. On day 7, the expressions of VEGF, TGF-*β*1, PDGF, EGF, and Collagen IV in the DYLG group were significantly higher than those in DC, DPJ, and DPC groups (Figures 5(c) and 5(d)). In contrast, the protein expression of MMP-9 was significantly lower than that in the other three groups.

On day 14, the levels of VEGF, TGF-*β*1, PDGF, EGF, and Collagen IV in the DYLG group were significantly higher than those in DC, DPJ, and DPC groups (*p* < 0.05), and the protein expression of MMP-9 was significantly lower than that in the DC group (Figures 5(e) and 5(f)). On day 21, the levels of VEGF, TGF-*β*1, PDGF, EGF, and Collagen IV were significantly higher in the DYLG group than those in DC (*p* < 0.05), and EGF and Collagen IV levels in the DYLG group were significantly higher than those in DPJ and DPC groups (*p* < 0.05). The protein expression of MMP-9 in DYLG group was significantly lower than that in DC, DPJ, and DPC groups (*p* < 0.05) (Figures 5(g) and 5(h)).

## 4. Discussion

Diabetes is a complex metabolic disease characterized by chronic hyperglycemia due to inadequate insulin secretion or insulin resistance. It is one of the critical factors impacting wound healing, which can cause a delay in wound healing, requiring hospitalization or possibly amputation. However, effective therapies are still limited in the clinic. This study has investigated that the Chinese medicinal formulation, Yeliangen (YLG), composed of *Coptidis rhizoma*, *Radix isatidis*, and *Isatidis folium*, can significantly promote wound healing by reducing complicated inflammation and increasing angiogenesis and collagen deposition. Notably, wound healing is associated with many chronic complications in individuals with diabetes, including long-term uncontrolled hyperglycemia, peripheral vascular disease, neuropathy, increased pressure at the wound site, and secondary infectious disease [34]. In this study, T2DM model SD rats were established by inducing diabetes with a high-fat diet and low-dose STZ to simulate the hyperglycemia status of diabetic rats [35, 36]. The results revealed that YLG could improve the impaired healing process by decreasing the inflammatory response and then enhancing angiogenesis. Whether YLG can improve other abnormal problems caused by diabetes, such as vascular disease and neuropathy, are worthy of further exploration and research by establishing appropriate animal or cell models.

The inflammatory stage is an essential stage of wound healing, and important proinflammatory factors such as TNF-*α*, IL-1B, and IL-6 are involved in this stage. The release of these proinflammatory cytokines is mediated by activating a transcription factor, NF-*κ*B, leading to an inflammatory response that recruits neutrophils and macrophages to the wound site [8]. High blood glucose has been reported to impede the function of these essential inflammatory mediators in wound healing. Furthermore, continuous inflammation causes a delay in the formation of granulation tissue and a failure to heal wounds of diabetes [37, 38]. Elevated levels of TNF-*α* and IL-1B have been revealed to increase cell apoptosis and decrease fibroblast proliferation, leading to impaired wound healing in diabetes [39]. Moreover, it was confirmed that MPP-9 could inhibit TGF-*β*1, and reducing the expression of MMP-9 can lead to the formation of extracellular matrix (ECM) [3, 40]. To assess the effect of YLG on persistent inflammation, we administered YLG twice daily at the wound site of diabetic rats. On various days, the expressions of TNF-*α*, NF-*κ*B, MMP-9, and IL-1B were determined in diabetic rats' wounded tissue. TNF-*α*, IL-1B, and MMP-9 levels on days 7, 14, and 21 postwounding were significantly reduced, indicating that YLG can effectively shorten the duration of the inflammatory wound phase in diabetic rats.

Endothelial cells and pericytes are recruited from existing blood vessels and proliferate in response to hypoxia, providing the essential oxygen and blood supply for tissue regeneration. This process is called neovascularization [20, 41]. Inadequate neovascularization is a critical characteristic of diabetic wound healing failure. Numerous investigations have been undertaken to determine the mechanism of this harm. Inadequate synthesis of angiogenic growth factors such as VEGF, TGF, EGF, and PDGF is widely regarded to be a primary cause of insufficient angiogenesis [13, 19, 42]. The results of this study showed that YLG could improve insufficient neovascularization in diabetic wound rats by enhancing endothelial cell and pericyte recruitment, which may be partly explained by the correction of angiogenic growth factors such as VEGF, TGF-*β*1, EGF, and PDGF deficiency. Moreover, TGF-*β*1 can recruit hematopoietic effector cells by activating VEGF expression through a variety of mechanisms that connect to neovascular pathways [43]. Previous studies have shown that VEGF and TGF-*β*1 levels were reduced in diabetic patients [13, 44]. PDGF plays an important role in the proliferation of fibroblasts and the production of collagen. *In vitro* study has explained the strong association between fibroblast proliferation and PDGF. This indicates that the application of PDGF nanoscaffolds has a longer activity duration than that of PDGF alone [18]. It has been demonstrated that increasing the expression of SDF-1*α* in diabetic wounds can normalize the production of therapeutic factors and restore their ability to promote angiogenesis [45]. YLG significantly increased the levels of VEGF, TGF-*β*1, EGF, PDGF, and SDF-1*α*, which supported suitable angiogenesis at the wounded tissues in the DYLG group. It has been established that VEGF administration can improve wound healing by enhancing angiogenesis and by mobilization and recruitment of bone marrow-derived cells [46]. In this study, the VEGF level increased on days 3, 7, and 14 but decreased on day 21 in the DYLG group; the reasonable reason for that may be the wounds in the early stages need VEGF for healing, but on day 21, the wound might heal completely, so no need for a high level of VEGF.

The MVD of the wound site increased in the DYLG group, which was confirmed by the IHC of CD31. Conversely, reduced MVD and inadequate angiogenesis in DC group led to poor wound healing. Thus, the utilization of YLG can improve angiogenesis, increasing the number of blood vessels and making them consistently distributed and well-formed. In previous studies, inhibition of eNOS expression or gene knockout has delayed wound healing, and significantly reduced eNOS expression in the skin of diabetic animals has been stated [47]. Therefore, in this study, the decreased expression of eNOS in the DC, DPJ, and DPC groups led to decreased and blocked angiogenesis; meanwhile, that was recovered in the DYLG group. IHC staining was used to determine the level of PCNA on the edge of the wound, and cell proliferation and regeneration were observed. Our experimental data showed that, compared with DC, DPJ, and DPC groups, PCNA expression was increased in the DYLG group, especially in fibroblasts and basal epidermis. These findings suggested that topical application of YLG can promote tissue regeneration and wound healing by increasing cell proliferation.

TGF-*β* also chemically attracts fibroblasts and enhances their proliferation [48]. Therefore, the higher level of TGF-*β*1 in the DYLG group may lead to significant fibroblast proliferation, which leads to more collagen deposition that is necessary for the granulation tissue formation and plays a key role in reepithelialization during the wound healing process [49]. In this study, western blotting and H&E showed that collagen expression increased in the DYLG group compared with DC, DPJ, and DPC groups. Moreover, TNF-*α* was significantly reduced, and TGF-*β*1 was significantly increased in the DYLG group, suggesting that YLG effectively regulated the ratio between TGF-*β*1 and TNF-*α* and promoted the formation of granulation tissue.

## 5. Conclusion

Wound healing impairment is one of the main complications of diabetes, and despite the accompanying hazards, therapeutic options for diabetic wounds stay limited, with no single effective medicine available to date. The results of this study revealed that the expressions of TNF-*α*, NF-*κ*B, MMP-9, and IL-1B were significantly decreased in the DYLG group compared with those in DC, DPJ, and DPC groups, while the levels of IL-10 were increased. YLG also increased the expression of VEGF, TGF-*β*1, PDGF, EGF, and SDF-1*α*. Moreover, in the DYLG group, granulation tissue was better, fibroblast proliferation was obvious, MVD was increased, collagen deposition was dense, and the epithelium regeneration was almost completed. The results showed that the wound healing effect in the DYLG group of diabetic rats was considerably improved. Therefore, YLG can significantly promote wound healing and be used as a prospective topical medicine to treat diabetic wounds.

## Figures and Tables

**Table 1:** The body weight/kg in T2DM rats on days 0, 3, 7, 14, and 21 (*n* = 24 per group).

Group	Day 0	Day 3	Day 7	Day 14	Day 21
DC	343.33 ± 13.42	349.67 ± 9.61	357.67 ± 3.18	366.00 ± 11.93	380.33 ± 15.62
DPJ	338.67 ± 20.30	340.33 ± 13.92	356.00 ± 8.14	365.67 ± 22.26	377.00 ± 22.68
DPC	337.33 ± 9.96	344.33 ± 15.07	352.00 ± 6.43	367.67 ± 17.13	369.33 ± 10.14
DYLG	328.00 ± 8.89	339.33 ± 7.33	345.33 ± 8.88	356.33 ± 13.04	351.67 ± 6.39^∗^^#¤^

DC: diabetic control group; DPJ: diabetic negative control group; DPC: diabetic positive control group; DYLG: diabetic Yeliangen-treated group. ^∗^*p* < 0.05 as compared to DC group, #*p* < 0.05 as compared to DPC group, and *^¤^p* < 0.05 as compared to DPJ group. The results were represented as mean ± SEM. ^∗^The meaning of the symbols in the following tables is the same in this study, so they will not be repeated.

**Table 2:** The serum glucose levels (mmol/l) in T2DM rats on days 0, 3, 7, 14, and 21 (*n* = 24 per group).

Group	Day 0	Day 3	Day 7	Day 14	Day 21
DC	22.01 ± 2.52	21.67 ± 3.38	20.33 ± 2.33	20.67 ± 1.20	20.67 ± 1.45
DPJ	21.67 ± 2.65	19.67 ± 2.33	19.33 ± 2.85	18.50 ± 2.08	18.17 ± 2.12
DP	21.66 ± 0.88	20.67 ± 1.20	20.67 ± 2.03	17.67 ± 0.88	18.00 ± 1.15
DYLG	21.33 ± 2.03	18.33 ± 0.88	18.00 ± 2.52	15.40 ± 0.60	13.40 ± 0.53^∗^^#¤^

**Table 3:** The blood insulin levels (ng/ml) in T2DM rats on days 0, 3, 7, 14, and 21 (*n* = 24 per group).

Group	Day 0	Day 3	Day 7	Day 14	Day 21
DC	0.31 ± 0.03	0.51 ± 0.07	0.76 ± 0.05	0.73 ± 0.06	0.81 ± 0.01
DPJ	0.32 ± 0.02	0.55 ± 0.06	0.75 ± 0.04	0.82 ± 0.04	0.82 ± 0.11
DPC	0.34 ± 0.05	0.56 ± 0.05	0.73 ± 0.05	0.81 ± 0.05	0.84 ± 0.07
DYLG	0.35 ± 0.05	0.54 ± 0.08	0.78 ± 0.04	0.96 ± 0.03^∗^	1.57 ± 0.02^∗^^,#,¤^

**Table 4:** The blood C-peptide levels (ng/ml) in T2DM rats on days 0, 3, 7, 14, and 21 (*n* = 24 per group).

Group	Day 0	Day 3	Day 7	Day 14	Day 21
DC	5.09 ± 0.33	4.83 ± 0.18	4.43 ± 0.25	4.25 ± 0.42	4.10 ± 0.07
DPJ	4.99 ± 0.33	4.94 ± 0.08	4.96 ± 0.08	4.95 ± 0.28	4.61 ± 0.24
DPC	5.07 ± 0.29	5.02 ± 0.15	4.96 ± 0.09	5.06 ± 0.25	5.32 ± 0.20
DYLG	5.11 ± 0.16	5.35 ± 0.23	5.57 ± 0.40	7.03 ± 0.49^∗^	7.24 ± 0.14^∗^^#,¤^

**Table 5:** The blood NF-*κ*B levels (ng/ml) in T2DM rats on days 0, 3, 7, and 14 (*n* = 24 per group).

Group	Day 0	Day 3	Day 7	Day 14	Day 21
DC	532.38 ± 17.91	3253.77 ± 202.07	2675.08 ± 239.31	2650.28 ± 299.92	2113.66 ± 215.21
DPJ	548.09 ± 2.81	2713.74 ± 171.03	2446.24 ± 57.45	2109.71 ± 31.44	1438.21 ± 61.58^∗^
DPC	514.23 ± 16.67	2456.64 ± 80.45^∗^	2154.90 ± 31.51	1850.86 ± 33.33^∗^	1355.86 ± 61.67^∗^
DYLG	556.18 ± 2.94	2426.64 ± 76.67^∗^	1362.17 ± 51.46^∗^^,#,¤^	1115.82 ± 21.85^∗^^,#,¤^	733.79 ± 40.15^∗^^,#,¤^

**Table 6:** The blood IL-6 level (ng/ml) in T2DM rats on days 0, 3, 7, 14, and 21 (*n* = 24 per group).

Group	Day 0	Day 3	Day 7	Day 14	Day 21
DC	54.47 ± 0.87	178.99 ± 0.88	118.36 ± 3.35	93.83 ± 1.29	86.31 ± 1.18
DPJ	54.80 ± 1.01	180.68 ± 0.58	101.61 ± 0.57	85.18 ± 0.57	79.89 ± 3.48
DPC	54.42 ± 0.83	177.77 ± 0.67	92.54 ± 1.53	81.88 ± 2.67	62.49 ± 1.15
DYLG	55.14 ± 0.58	168.43 ± 0.58	71.61 ± 0.57^∗^^,#,¤^	62.88 ± 0.58^∗^^,#,¤^	52.54 ± 1.53^∗^^,#,¤^

## Data Availability

The data supporting the findings of this study are accessible upon request from the corresponding author.

## Supplementary Materials

Supplementary Materials Table S1: The experimental reagents.

## References

[1] Tan S. Y., Wong J. L. M., Sim Y. J. (2019). Type 1 and 2 diabetes mellitus: a review on current treatment approach and gene therapy as potential intervention. *Diabetology & Metabolic Syndrome*.

[2] Saeedi P., Petersohn I., Salpea P. (2019). Global and regional diabetes prevalence estimates for 2019 and projections for 2030 and 2045: results from the International Diabetes Federation Diabetes Atlas, 9 th edition. *Diabetes Research and Clinical Practice*.

[3] Kant V., Kumar D., Kumar D. (2015). Topical application of substance P promotes wound healing in streptozotocin-induced diabetic rats. *Cytokine*.

[4] Robert F., Franks P., Edmonds M. (2020). A multinational, multicenter, randomized, double-blinded, placebo-controlled trial to evaluate the efficacy of cyclical topical wound oxygen (TWO2) therapy in the treatment of chronic diabetic foot ulcers: the TWO2 study. *Diabetes Care*.

[5] Tong T., Yang C., Tian W. (2020). Phenotypes and outcomes in middle-aged patients with diabetic foot ulcers: a retrospective cohort study. *Journal of Foot and Ankle Research*.

[6] Nguyen C., Tartar D., Bagood M. (2019). Topical fluoxetine as a novel therapeutic that improves wound healing in diabetic mice. *Diabetes*.

[7] Zubair M., Fatima F., Zubair M., Ahmad J., Malik A., Talluri M. R. (2021). Molecular mechanism and biomechanics of the diabetic foot: the road to foot ulceration and healing. *Diabetic Foot Ulcer*.

[8] Kandhare A., Ghosh P., Bodhankar S. (2014). Naringin, a flavanone glycoside, promotes angiogenesis and inhibits endothelial apoptosis through modulation of inflammatory and growth factor expression in diabetic foot ulcer in rats. *Chemico-Biological Interactions*.

[9] Andrew P., Dave H. (2020). *Physiology, Hemostasis*.

[10] Xiao J., Li J., Cai L., Chakrabarti S., Li X. (2014). Cytokines and diabetes research. *Journal of Diabetes Research*.

[11] Falanga V. (2015). Wound healing and its impairment in the diabetic foot. *The Lancet*.

[12] Veith A. P., Henderson K., Spencer A., Sligar A. D., Baker A. B. (2019). Therapeutic strategies for enhancing angiogenesis in wound healing. *Advanced Drug Delivery Reviews*.

[13] Okonkwo U., Chen L., Ma D. (2020). Compromised angiogenesis and vascular integrity in impaired diabetic wound healing. *PLoS One*.

[14] Sharma S., Schaper N., Rayman G. (2019). Microangiopathy: is it relevant to wound healing in diabetic foot disease?. *Diabetes/Metabolism Research and Reviews*.

[15] Romero-Cerecero O., Zamilpa A., Díaz-García E., Tortoriello J. (2014). Pharmacological effect of Ageratina pichinchensis on wound healing in diabetic rats and genotoxicity evaluation. *Journal of Ethnopharmacology*.

[16] Ko U., Choi J., Choung J., Moon S., Shin J. H. (2019). Physicochemically tuned myofibroblasts for wound healing strategy. *Scientific Reports*.

[17] Asiri A., Saidin S., Sani M. H., al-Ashwal R. H. (2021). Epidermal and fibroblast growth factors incorporated polyvinyl alcohol electrospun nanofibers as biological dressing scaffold. *Scientific Reports*.

[18] Robert S. (2020). Nanotechnology in the future treatment of diabetic wounds. *The Review of Diabetic Studies*.

[19] Zubair M., Ahmad J. (2019). Role of growth factors and cytokines in diabetic foot ulcer healing: a detailed review. *Reviews in Endocrine & Metabolic Disorders*.

[20] Chen L., Zheng Q., Liu Y. (2020). Adipose-derived stem cells promote diabetic wound healing via the recruitment and differentiation of endothelial progenitor cells into endothelial cells mediated by the VEGF-PLC*γ*-ERK pathway. *Archives of Biochemistry and Biophysics*.

[21] Spampinato S., Caruso G., De Pasquale R., Sortino M. A., Merlo S. (2020). The treatment of impaired wound healing in diabetes: looking among old drugs. *Pharmaceuticals*.

[22] Mahboubi M., Taghizadeh M., Khamechian T., Tamtaji O. R., Mokhtari R., Talaei S. A. (2018). The wound healing effects of herbal cream containing *Oliveria decumbens* and *Pelargonium graveolens* essential oils in diabetic foot ulcer model. *World Journal of Plastic Surgery*.

[23] Karri V., Kuppusamy G., Talluri S., Yamjala K., Mannemala S. S., Malayandi R. (2016). Current and emerging therapies in the management of diabetic foot ulcers. *Current Medical Research and Opinion*.

[24] Zhou X., Guo Y., Yang K., Liu P., Wang J. (2022). The signaling pathways of traditional Chinese medicine in promoting diabetic wound healing. *Journal of Ethnopharmacology*.

[25] Wang A., Yuan Q., Guo N., Yang B., Sun Y. (2021). Research progress on medicinal resources of Coptis and its isoquinoline alkaloids. *China Journal of Chinese Materia Materia Medica*.

[26] Jiang L., Yu Y., Jin J. (2015). *n*-Butanol extract from Folium isatidis inhibits lipopolysaccharide-induced inflammatory cytokine production in macrophages and protects mice against lipopolysaccharide-induced endotoxic shock. *Drug Design, Development and Therapy*.

[27] Nie L., Wang X., Huang L. (2022). Construction of information bank of chemical components in Isatidis radix. *Chinese Pharmaceutical Journal*.

[28] Fan Z., Cai L., Wang Y., Zhu Q., Wang S., Chen B. (2021). The acidic fraction of Isatidis radix regulates inflammatory response in LPS-stimulated RAW264.7 macrophages through MAPKs and NF-*κ*B pathway. *Evidence-Based Complementary and Alternative Medicine*.

[29] Yan J., Gu J., Feng S., Ji R. F., Quan Q. H., Liu Y. G. (2019). Study of chemical constituent and antioxidant activity of Isatids folium. *Journal of Chinese Mass Spectrometry Society*.

[30] Dardmah F., Farahpour M. R. (2021). Quercus infectoriagall extract aids wound healing in a streptozocin-induced diabetic mouse model. *Journal of Wound Care*.

[31] Talebi H., Farahpour M. R., Hamishehkar H. (2021). The effectiveness of rutin for prevention of surgical induced endometriosis development in a rat model. *Scientific Reports*.

[32] Habibi Zadeh S. K., Farahpour M. R., Kar H. H. (2020). The effect of topical administration of an ointment prepared from Trifolium repens hydroethanolic extract on the acceleration of excisional cutaneous wound healing. *Wounds*.

[33] Kunkemoeller B., Bancroft T., Xing H. (2019). Elevated thrombospondin 2 contributes to delayed wound healing in diabetes. *Diabetes*.

[34] Patel S., Srivastava S., Singh M., Singh D. (2019). Mechanistic insight into diabetic wounds: pathogenesis, molecular targets and treatment strategies to pace wound healing. *Biomedicine and Pharmacotherapy*.

[35] Lan C., Wu C., Huang S., Wu I. H., Chen G. S. (2013). High-glucose environment enhanced oxidative stress and increased interleukin-8 secretion from keratinocytes. *Diabetes*.

[36] Mishra A., Yedella K., Lakshmi J., Siva A. B. (2018). Wdr13 and streptozotocin-induced diabetes. *Nutrition & Diabetes*.

[37] Jain M., LoGerfo F., Guthrie P., Pradhan L. (2011). Effect of hyperglycemia and neuropeptides on interleukin-8 expression and angiogenesis in dermal microvascular endothelial cells. *Journal of Vascular Surgery*.

[38] Li M., Yu H., Pan H. (2019). Nrf2 suppression delays diabetic wound healing through sustained oxidative stress and inflammation. *Frontiers in Pharmacology*.

[39] Yang H., Fierro F., So M. (2020). Combination product of dermal matrix, human mesenchymal stem cells, and timolol promotes diabetic wound healing in mice. *Stem Cells Translational Medicine*.

[40] Wang W., Yang C., Wang X. (2018). MicroRNA-129 and -335 promote diabetic wound healing by inhibiting Sp1-mediated MMP-9 expression. *Diabetes*.

[41] Kaushik K., Das A. (2020). TWIST1-reprogrammed endothelial cell transplantation potentiates neovascularization-mediated diabetic wound tissue regeneration. *Diabetes*.

[42] Jeong S., Kim B., Park M., Ban E., Lee S. H., Kim A. (2020). Improved diabetic wound healing by EGF encapsulation in gelatin-alginate coacervates. *Pharmaceutics*.

[43] Fang S., Pentinmikko N., Ilmonen M., Salven P. (2012). Dual action of TGF-*β* induces vascular growth *in vivo* through recruitment of angiogenic VEGF-producing hematopoietic effector cells. *Angiogenesis*.

[44] Al-Mulla F., Leibovich S., Francis I., Bitar M. S. (2011). Impaired TGF-*β* signaling and a defect in resolution of inflammation contribute to delayed wound healing in a female rat model of type 2 diabetes. *Molecular BioSystems*.

[45] Laiva A., O’Brien F., Keogh M. (2021). SDF-1*α* gene-activated collagen scaffold restores pro-angiogenic wound healing features in human diabetic adipose-derived stem cells. *Biomedicine*.

[46] Galiano R., Tepper O., Pelo C. (2004). Topical vascular endothelial growth factor accelerates diabetic wound healing through increased angiogenesis and by mobilizing and recruiting bone marrow- derived cells. *The American Journal of Pathology*.

[47] Song M., Chen L., Zhang L. (2020). Cryptotanshinone enhances wound healing in type 2 diabetes with modulatory effects on inflammation, angiogenesis and extracellular matrix remodelling. *Pharmaceuitical Biology*.

[48] Mokoena D. R., Houreld N. N., Dhilip Kumar S. S., Abrahamse H. (2020). Photobiomodulation at 660 nm stimulates fibroblast differentiation. *Lasers in Surgery and Medicine*.

[49] Long G., Liu D., He X. (2020). A dual functional collagen scaffold coordinates angiogenesis and inflammation for diabetic wound healing. *Biomaterials Science*.

