# Supplementary material for: Topical Application of Chinese Formula Yeliangen Promotes Wound Healing in Streptozotocin-Induced Diabetic Rats
Source: J Diabetes Res. 2022 Nov 29;2022:1193392. doi: 10.1155/2022/1193392 (PMC9726244; doi:10.1155/2022/1193392)
Supplement: Supplementary Materials — Table S1: The experimental reagents. [file 1193392.f1.docx]

**Supplementary description:**

**Table S1: The experimental reagents**

| **Name of reagent** | **Batch number** | **Manufacturer** |
| --- | --- | --- |
| Radioimmunoprecipitation assay buffer (RIPA) | P0013B | Shanghai Beyotime Company |
| Phenylmethylsulfonyl fluoride（PMSF） | ST506 | Shanghai Beyotime Company |
| BCA Protein Concentration Assay Kit | P0010S | Shanghai Beyotime Company |
| β-actin Antibody | 4970S | Cell Signaling Technology |
| INS ELISA kit | MTSNKEVHNZ | Wuhan Biotechnology Co., LTD |
| C-peptide ELISA kit | E-EL-R0032c | Wuhan Biotechnology Co., LTD |
| NF-κB) ELISA kit | E-EL-R0674c-96T | Wuhan Biotechnology Co., LTD |
| IL-6 ELISA kit | E-EL-R0015C-96T | Wuhan Biotechnology Co., LTD |
| VEGF Antibody | 19003-1-AP- | Proteintech Group |
| TGF- β 1 Antibody | 21898-1-AP- | Proteintech Group |
| PDGF-D Antibody | Abp53030 | Biotechnology Co., LTD |
| Collagen IV Antibody | Abp57493 | Biotechnology Co., LTD |
| MMP-9 Antibody | Abp51421 | Biotechnology Co., LTD |
| EGF Antibody | ab184265-40ul | Abcam Company |
| PVDF membranes | \ | Thermo Fisher Scientific, Millipore, MA |
| SDS-PAGE loading buffer（5X） | P0015 | Shanghai Beyotime Company |
| SDS-PAGE Gel preparation Kit | P0012A | Shanghai Beyotime Company |
| BeyoColor™ Prestained Color Protein Marker（10-170kD） | P0075 | Thermo Fisher Scientific |
| SDS-PAGE Running Buffer Tris | T1070 | Beijing Solarbio Science and technology Co. |
| 10× Electrophoretic transfer buffer (membrane transfer solution) | D1060 | Beijing Solarbio Science and technology Co. |
| Western solution set (block solution, wash solution, primary antibody and secondary antibody diluents） | P0023 | Shanghai Beyotime Company |
| Anti-rabbit IgG（H+L）（DyLight™ 680 Conjugate） | AP0614 | Cell Signaling Technology |
| Total RNA extraction kits | 03918KD1 | Axygen Biotechnology Co., Ltd, USA |
| PowerUpTM SYBRTM Green Mater Mix | A25742 | Thermo Fisher Scientific Co., LTD |
| The PrimeScripTM RT Reagent Kit | RR036A-1 | Takara Bio. Japan |
| Streptozotocin (STZ) | S8050-1g | Solarbio Science & Technology Co., Ltd |
